# Large Hair Ball

**Published:** 1915-05

**Authors:** 


**Large Hair Ball.** Matas, N. O. Med. & Surg. Jour. describes a case in a girl aged 19 who had acquired the habit of trichophagy as a child while suffering from uncinariasis. The mass weighed 967 grams, and almost completely filled the stomach, extending through the pylorus into the duodenum. Recovery after gastrotomy.

